# Effects of carbon dioxide accumulation on post-dive physiological recovery in odontocetes

**DOI:** 10.1242/jeb.251853

**Published:** 2026-04-13

**Authors:** Emily C. Nazario, Max F. Czapanskiy, Malin L. Pinsky, Dennis R. Christen, Katherine L. Flammer, Kelsey A. Ford, Traci L. Kendall, Bryan Tom, Sam Bartosik-Velez, Joshua Allyn, Fanny R. Sánchez Villarreal, Terrie M. Williams

**Affiliations:** ^1^ University of California, Santa Cruz, Ecology and Evolutionary Biology Department, 130 McAllister Way, Santa Cruz, CA 95060, USA; ^2^ College of Creative Studies, University of California, Santa Barbara, 1 UCEN Rd, Santa Barbara, CA 93106, USA; ^3^ Bren School of Environmental Science & Management, University of California, Santa Barbara, Santa Barbara, CA 93117, USA; ^4^ Georgia Aquarium, 225 Baker St NW, Atlanta, GA 30313, USA; ^5^ Arizona State University, Conservation Biology and Ecology, 1151 S Forest Ave, Tempe, AZ 85281, USA

**Keywords:** Dive recovery, Bottlenose dolphins, Beluga whales, Blood acidosis, Breathing patterns, Peripheral vasodilation

## Abstract

Diving performance by marine mammals is associated with marked changes in tissue oxygen (O_2_) and carbon dioxide (CO_2_) levels. Yet, the primary metric for diving recovery in most studies has focused exclusively on restoring tissue O_2_, despite the importance of CO_2_ offloading as a major determinant for diving homeostasis. To assess the combined role of respiratory and blood gases, we compared post-exercise O_2_ and CO_2_ recovery rates in bottlenose dolphins (*Tursiops truncatus*, *n*=2) and beluga whales (*Delphinapterus leucas*, *n*=4). System-wide recovery mechanisms were also examined, including blood pH, breathing patterns and peripheral vasodilation. Following maximal swim repetitions, respiratory O_2_ and CO_2_ rates returned to resting levels within 8 min for belugas (*V̇*_O_2__: 7.64±1.36 min; *V̇*_CO_2__: 7.71±1.41 min; mean±s.d.) and 3.5 min for dolphins (*V̇*_O_2__: 3.41±0.76 min; *V̇*_CO_2__: 3.41±0.71 min). Blood O_2_ and CO_2_ recovery durations also varied by species. Belugas required 12–15 min to reach resting levels, whereas dolphins' blood O_2_ remained within resting levels and CO_2_ recovered in ∼4–7 min. Blood pH, driven by changes in *P*_CO_2__, returned to resting levels between 12 and 15 min for belugas, but remained elevated throughout the recorded recovery period for dolphins. Blood lactate also remained near double the resting values for both species. Overall, we found that the compounding effects of CO_2_ with blood lactate appear to play a dominant role in odontocete dive recovery, which will dictate the duration of full physiological recovery by wild odontocetes following escape responses from anthropogenic disturbances.

## Teaser

**Summary:** Carbon dioxide accumulation and whole-body recovery timelines following sequential swimming for two odontocetes indicate that blood pH, lactate and changes in peripheral perfusion may represent key metrics that prolong whole-body dive recovery.

## INTRODUCTION

Diving by wild marine mammals consists of dozens to hundreds of sequential dives per day. To maintain a daily cycle of extended apnea interspersed with brief recovery breathing periods at the surface, diving mammals use a suite of adaptations acquired over 50 million years of evolution that allow them to withstand rapid, marked fluctuations in blood oxygen (O_2_) and carbon dioxide (CO_2_) (Butler, 1982; Lenfant et al., 1970; Noren, 2004). Although considerable research has been conducted on the use of O_2_ stores by diving marine mammals, far less is known about the simultaneous effects of CO_2_ accumulation during submergence or its offloading during the post-dive recovery period (Boutilier et al., 2001; Butler, 1982; Noren et al., 2012a). This has led to an emphasis on recharging O_2_ stores and removing anaerobic metabolic byproducts during surface recovery periods in preparation for subsequent dives (Génin et al., 2015; Kooyman et al., 1980). This is despite suggestions that perturbations in CO_2_ stores may represent a bottleneck determining when diving can resume (Boutilier et al., 2001).

Previous studies have reported marked changes in physiological homeostasis and diving behavior associated with elevated CO_2_, including (1) altering body responses to depleted O_2_ stores, (2) signaling dive termination and (3) extending dive recovery times (Brooks and Gladden, 2003; Brubakk et al., 2007; Fahlman et al., 2019; Rosen et al., 2017). Elevated blood CO_2_ concentrations, plasma lactate levels, and associated changes in blood pH can also significantly affect tissue recovery; all are considered major factors contributing to exercise fatigue and extended recovery times (Boutilier et al., 2001; Brooks and Gladden, 2003; Brubakk et al., 2007). In terrestrial mammals, changes in blood pH following CO_2_ accumulation and acidosis can result in reduced muscle contractile ability, muscle fatigue and cell/tissue/organ damage, leading to symptoms such as headaches, anxiety and delirium (Brooks and Gladden, 2003; Cummins et al., 2020; Handy and Soni, 2008).

Numerous physiological processes help maintain homeostasis by counteracting potential detrimental effects of increased tissue CO_2_ in exercising mammals. For example, marine mammals undergo extended apneas and display substantially higher CO_2_ and pH buffering capacities compared with terrestrial animals of similar size (Castellini and Somero, 1981; Lenfant et al., 1970; Noren, 2004). Underlying mechanisms include many of the same adaptations contributing to O_2_ storage and diving capacities, such as comparatively large blood volumes and elevated red blood cell concentrations, that enhance CO_2_ transport and removal (Boutilier et al., 1993; Davis, 2014, 2019). However, the deepest diving cetaceans with the highest blood buffering capacities may demonstrate high levels of acidic metabolic byproducts and subsequent recovery behaviors (e.g. extended surface periods, sequential short and shallow dives) following exceptionally prolonged dives or escape maneuvers (Noren, 2004; Quick et al., 2020; Williams et al., 2017a). Specifically, events that trigger flight responses, such as seismic airguns and vessel noise, may alter sequential diving patterns, thereby disrupting normal dive recovery times (Williams et al., 2017b). Boutilier et al. (2001) has also suggested that marine mammals may postpone complete recovery to take advantage of valuable foraging opportunities, momentarily ‘accepting’ CO_2_ accumulation and subsequent changes in blood pH for short periods. Thus, high-intensity exercise behaviors likely skew recovery timelines for O_2_ and CO_2_, and the return to homeostasis for systems involved in their transport (e.g. ventilation, vasculature). Ultimately, this will determine diving ability.

**Fig. 1.**
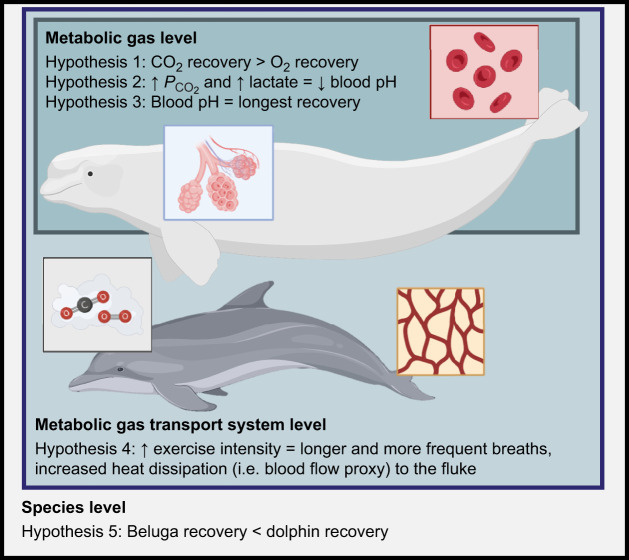
**Overview of study hypotheses.** Summary of the hypotheses grouped by their corresponding level of organization (metabolic gas, transport system and species level). Icons and images are from Pixabay and BioRender. Created in BioRender by Nazario, E., 2026. https://BioRender.com/zcgsc8s. This figure was sublicensed under CC-BY 4.0 terms.

To further understand these processes, we conducted a comparative analysis to document the integrative recovery timelines of metabolic, ventilatory and peripheral perfusion processes following exercise varying in intensity. Both repetitive swimming and surface-active behaviors (SABs; e.g. bows, spins and rolls) were examined. The timing and patterns of swims and SABs were based on observed behaviors in the wild. Typically, these offer limited recovery opportunities, which instigate CO_2_ accumulation. Owing to the high solubility of CO_2_ stored in tissues, we hypothesized that respiratory and blood CO_2_ would require longer recovery durations compared with O_2_ (Fig. 1; Boutilier et al., 2001; Fahlman et al., 2008; Gerlinsky et al., 2014; Rosen et al., 2017). Further, we expected that blood pH would remain below resting levels (i.e. more acidic) longer than the changes observed in blood O_2_ and CO_2_. This could be driven by the compounding and complex relationship between lactic acid and CO_2_ accumulation in the tissues requiring longer readjustment periods (Kooyman et al., 1980; Stringer et al., 1992). We also predicted that breathing pattern and peripheral perfusion recovery would correspond with increasing exercise intensity to support rapid O_2_ onboarding and CO_2_ offloading (Favilla et al., 2022; McArdle et al., 1957; Meir and Ponganis, 2010; Noren et al., 2012b; Shaffer et al., 1997; Williams et al., 1993). To investigate how recovery timelines differ among odontocetes with different body masses and diving adaptations, we examined two species, 200 kg bottlenose dolphins (*Tursiops truncatus*) with sprint speeds of 8.3 m s^−1^ and maximal swim durations of 8 min, and 1400 kg beluga whales (*Delphinapterus leucas*) that can breath-hold for 18 min and sprint at 1.6 m s^−1^ (Noren, 2004). Larger body sizes, longer dive durations and tissue-level adaptations for beluga whales (e.g. higher myoglobin concentrations, increased muscle buffering capacities compared to dolphins) were also expected to shorten recovery times across all metrics relative to dolphins (Noren, 2004; Noren and Williams, 2000). Together, these tests provide a comparative analysis of recovery timelines for metabolic gases and their transport systems, which support high levels of exercise associated with intense foraging activity or flight responses due to anthropogenic disturbance.

## MATERIALS AND METHODS

### Animals, facilities and experimental design

Two adult male bottlenose dolphins [*Tursiops truncatus* (Montagu 1821); ages 8–16 years], three adult female beluga whales [*Delphinapterus leucas* (Pallas 1776); ages 15–26 years] and one adult male beluga whale (age 14–16 years) participated in these trials (Table S1). The odontocetes were housed in saltwater pools maintained at 20–21.5°C (dolphins, University of California, Santa Cruz, Santa Cruz, CA, USA) and 15°C (belugas, Georgia Aquarium, Atlanta, GA, USA). Maximum pool depths were 9.1 m for dolphins and 7.3 m for belugas. Air temperatures remained between 12°C and 27.9°C for dolphins and between 16.8°C and 20.1°C for belugas. Morphometric measurements (body mass, girths, photogrammetry, etc.) were taken to assess body condition. Both species were fed multiple times per day on a mixed fish diet supplemented with vitamins consistent with maintaining normal seasonal and growth patterns, while accounting for age class and hormonal state. Although body masses may fluctuate for individuals depending on their status, body condition was kept at a healthy range for each animal. Data collection took place between August 2021 and April 2025, and training for exercise (swims, SABs) and resting behaviors was completed prior to data collection. All procedures were approved by the University of California, Santa Cruz Institutional Animal Care and Use Committee (protocol no. Willt2302dn). This study was conducted under a Marine Mammal Protection Act permit issued by the NOAA Fisheries Office of Protected Species (permit no. 24054).

**Fig. 2.**
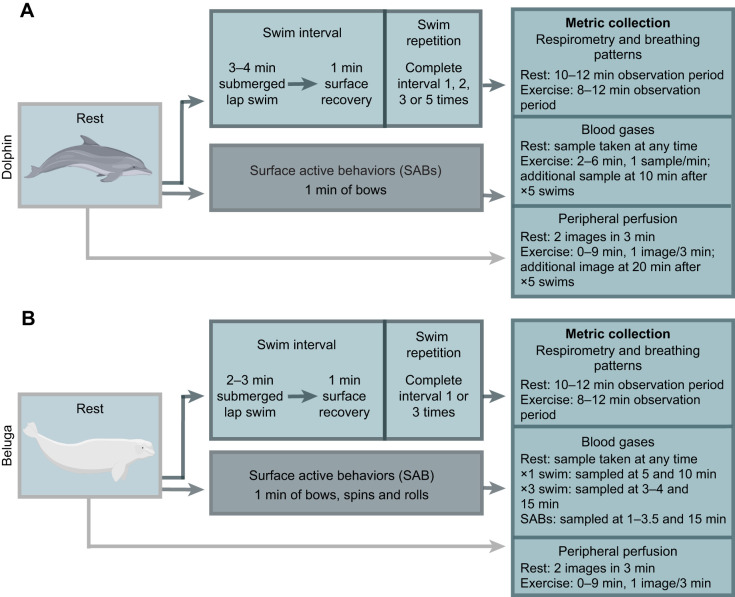
**Schematic of experimental design.** Overview of the exercises completed by (A) dolphins and (B) belugas. Metric collection timelines [respirometry and breathing patterns, blood gases and forward-looking infrared (FLIR) images] are also summarized per species. Metric collection times are further separated by trial type when collection timing varied. Images from Pixabay and BioRender. Created in BioRender by Nazario, E., 2026. https://BioRender.com/lgtisoi. This figure was sublicensed under CC-BY 4.0 terms.

Metrics (see sub-sections below) were recorded at rest, following repetitive submerged swims, and following 1 min of high-intensity SABs, which consisted of bows (dolphins) or a mix of bows, spins and rolls (belugas) (Fig. 2; Table S1). All metrics were measured during separate trials on separate days, except for respirometry and breathing patterns, which were recorded simultaneously. Resting measurements consisted of the animal voluntarily floating upright on the water's surface. The animals completed standardized repetitive submerged lap swims >1–2 m below the surface in a measured loop circuit (dolphin: 59 m lap length, beluga: 34 m lap length) for 3–4 min (dolphins) or 2–3 min (belugas) followed by a 1 min period at the surface. The ‘3 min swim–1 min surface’ interval trials were repeated one to three times for belugas or five times for dolphins. Each replicate swim count (one, two, three or five) was randomly assigned to a day throughout the data collection period. The timing of these swim interval protocols was based on the reported swimming responses of wild odontocetes following human disturbance (Cascão, 2001; Lerczak et al., 2000; Sivle et al., 2012). The 1 min surface duration allowed the animals to temporarily recover while preventing a complete return to rest (Fahlman et al., 2008).

Swim speeds reflected the preferred voluntary speed for each animal at relatively constant stroking. To assess speed consistency across swim repetitions, lap speed was recorded. For a subset of trials, we also monitored acceleration as recorded with a tri-axial accelerometer tag to provide an additional metric to quantify and ensure performance consistency across swims (Cats Diary, 400 Hz, Custom Animal Tracking Solutions, Moffat Beach, Queensland, Australia) (Fig. S1). The 1 min of voluntary SABs were used to represent maximal energetic output per animal (Noren et al., 2012a). Dolphins completed an average of 16.4 bows (*n*=13 trials), whereas belugas completed a mix of SAB activities during the 1 min test period (*n*=15 trials).

### Respirometry, breathing patterns, blood gases and vasculature metrics

#### Respirometry and breathing patterns

Mass-specific oxygen consumption (*V̇*_O_2__) and carbon dioxide production (*V̇*_CO_2__) rates were measured using open-flow respirometry following the protocols of Williams et al. (2004, 2017a) (Fig. S2). The most recent available body weights for each animal were used. Animals were trained to rest while breathing under a plexiglass metabolic hood (hereafter, ‘dome’; dolphin: 85×58×36 cm; beluga: 127×81×36 cm, length×width×height) mounted on a PVC frame that rested on the water's surface. Air was pulled through the dome with a calibrated vacuum pump (FlowKit Mass Flow Generator, Sable Systems International, North Las Vegas, NV, USA) at a rate of 300 l min^−1^ (dolphin) and 500 l min^−1^ (beluga). The rate of airflow was regulated and subsampled at 400–500 ml min^−1^ (both species) using a metabolic gas analyzer (Field Metabolic System, Sable Systems). Prior to gas measurements, the subsample was passed through three tubes filled with desiccant (Drierite, W. A. Hammond Drierite, Xenia, OH, USA), and prior to oxygen measurements, the subsample was passed through two additional tubes filled with a CO_2_ absorbent (Sodasorb, W.R. Grace & Co., Columbia, MD, USA). Subsampled metabolic gases were continuously monitored and recorded at 1 Hz on a laptop computer using ExpeData Analysis software (Sable Systems). These values were then corrected for standard temperature and pressure and converted to *V̇*_O_2__ and *V̇*_CO_2__ using equations from Lighton (2018) and Withers (1977). The metabolic gas analyzer was corrected each day using dry ambient air (20.95% O_2_) and calibrated at the start of the overall study period using N_2_ gas and carbon dioxide (5% CO_2_, 95% N_2_). Calibrations were performed using previously described protocols (Davis et al., 1985; Fedak et al., 1981; Withers, 1977). The three desiccant tubes were in the sample path whereas the ambient oxygen corrections were performed before data collection. Desiccant tubes were removed from the sample path prior to data collection to avoid distortion of CO_2_ measurements. Effects of water vapor pressure were mathematically corrected (Lighton, 2018).

Dolphins and belugas completed rest or exercise sessions while fasted (>8 h since the last feeding). The number of trials for dolphins were: *n*_rest_=15, *n*_×1swim_=12, *n*_×3swim_=8, *n*_×5swim_=10, *n*_SAB_=4. The number of trials for belugas were: *n*_rest_=4, *n*_×3swim_=5, *n*_SAB_**=**5. Metabolic rates were measured for both dolphins and two of the adult female beluga whales (Table S1). For resting measurements, the animal floated next to the dome for 1–2 min to minimize any additional energetic costs from traveling to the dome (John et al., 2024). They then moved into the dome, where they rested dorsal side up for 10–12 min in a stationary position (Fig. 2). For the exercise sessions, the animals would complete the requested behavior or series of behaviors, immediately followed by stationing under the dome. The animals remained resting in this stationary position for 8–12 min. The duration of recovery in the dome was determined by the return of the animal's O_2_ and CO_2_ saturation percentages to near resting levels as measured previously (Fig. S2).

Respiratory gases and breathing patterns were measured both simultaneously and in extra session for dolphins (*n*_rest_=19, *n*_×1swim_=12, *n*_×3swim_=8, *n*_×5swim_=10, *n*_SAB_=4) and belugas (*n*_rest_=3, *n*_×3swim_=5, *n*_SAB_**=**5). Breathing patterns were videorecorded with a GoPro camera (GoPro Hero 5, GoPro, San Mateo, CA, USA) set to a resolution of 1920×1080 pixels and 30 Hz frame rate. Breath duration and inter-breath interval (IBI) were assessed. We defined breath duration as the period between the start of the exhalation through the end of the corresponding inhalation. IBI was the period between the end the last breath and the start of the next breath, or the end of an inhalation and the start of the subsequent exhalation for cetaceans (Isojunno et al., 2018). Breathing frequency (breaths min^−1^) was also determined from taking the inverse of the IBI (seconds between breaths) (Fahlman et al., 2016). Importantly, the IBI was the period between breaths rather than the period between breath cycles, so we would expect breath frequency values to slightly vary if calculated from the inverse of breath cycle durations. Breathing pattern metrics were recorded following the final swim or SAB of the session while the animal was under the metabolic dome. Videos were analyzed using Behavioral Observation Research Interactive Software (BORIS; Friard and Gamba, 2016). Frame-by-frame mode was used to identify when the blowhole opened (start of the exhale) and closed (end of the inhale). Breaths where glare or condensation accumulated on the metabolic dome's interior were omitted from the study (13.5% of the ventilation dataset pooled across species).

#### Blood gases

Owing to the interference of the metabolic hood, blood samples were collected in separate sessions from the respiratory tests. Partial pressures of oxygen and carbon dioxide (*P*_O_2__ and *P*_CO_2__; mmHg), pH, lactate (mmol l^−1^), bicarbonate (HCO_3_^−^; mmol l^−1^) and Hb–O_2_ saturation (*S*_O_2__; %) were determined from whole-blood samples (mix of arterial and venous) collected from the fluke following each trial type (rests, sequential swims, SABs). Both dolphins and all beluga whales participated in blood collection, but not every beluga was used in each trial type (Table S1) (dolphin: *n*_rest_=4, *n*_×1swim_=16, *n*_×3swim_=16, *n*_×5swim_=20, *n*_SAB_=4; beluga: *n*_rest_=6, *n*_×1swim_=21, *n*_×3swim_=9, *n*_SAB_=8). Animals were fasted prior to blood collection (>8 h since the last feeding) but were fed once the session started. Blood samples were collected within 30 min after receiving food to minimize changes in metabolic rates following a feeding event. A 21 gauge, ¾ inch butterfly catheter (Vaculet Blood Collection Set, Excelint International Company, Redondo Beach, CA, USA) was placed in the periarterial venous rete, using the vascular groove on the fluke's ventral face as a blood collection target (Geraci and Lounsbury, 2005). Whole blood was collected into a 3 ml sodium heparin syringe (Line Draw Arterial Blood Sampling Kit with Dry Lithium Heparin for Gases and Electrolytes, Smiths Medical ASO, Keene, NH, USA). For dolphins, samples were collected at 1 min timesteps between 2 and 6 min following the final swim or SABs (Fig. 2). Additional samples were taken at 10 min following the final swim of the ×5 sequential swims. To accommodate their larger size and prolonged recovery period (see respiratory gas results), the timing for blood sampling was scaled up for the beluga whales. Samples were collected at 5 and 10 min following a single 3 min swim, between 3 and 4 min and at 15 min following three sequential swims, and between 1 and 3.5 min and at 15 min following the SABs.

All samples were analyzed with a portable blood gas analyzer (i-STAT 1, Abbott Laboratories, Abbott Park, IL, USA) which has been calibrated and extensively tested for use with marine mammal blood, including bottlenose and common dolphins (*Delphinus delphis*) (Flower et al., 2018; Sharp et al., 2014). Within 10 min of blood collection, 3–5 drops of blood were placed into cartridges that measured blood gas metrics (CG4+ i-STAT cartridges, Abbott Laboratories, Abbott Park, IL, USA). Values were determined within 3 min of being inserted into the analyzer. Precision data for these cartridges and analyzer are available for blood pH (±0.01, 95% CI), *P*_O_2_ _(±6.24 mmHg, 95% CI), *P*_CO_2_ _(±3.14 mmHg, 95% CI) and lactate (±0.16 mmol l^−1^, 95% CI).

#### Forward-looking infrared (FLIR) imaging

Both dolphins and all beluga whales participated in thermal image collection, although not every beluga was used for each trial type (dolphin: *n*_rest_=10, *n*_×1swim_=11, *n*_×3swim_=10, *n*_×5swim_=12, *n*_SAB_=4; beluga: *n*_rest_=20, *n*_×1swim_=8, *n*_×3swim_=6, *n*_SAB_=6) (see Table S1). The odontocetes were trained to present their flukes voluntarily for thermal image collection. At rest, images were taken using a FLIR camera in two bouts separated by 3 min and then averaged (Fig. 2). For repetitive swimming and SAB sessions, images were taken immediately after the activity and then at 3 min time intervals for 9 min post-exercise. For the dolphins, additional images were taken at 20 min following the final swim of the ×5 sequential swim. Between image collection, animals remained resting at station in a low energy state.

Prior to these thermal sessions, a calibration experiment was completed to assess the accuracy and precision of the FLIR camera (FLIR C5, Teledyne FLIR, Wilsonville, OR, USA). Skin temperatures were recorded from the ventral surface of the dolphin's fluke using a handheld infrared temperature sensor (Nubee Infrared Thermometer, Nubee, Rancho Cucamonga, CA, USA) alongside of the FLIR camera. Temperature measurements from both devices were compared, and the camera remained within 2°C of the handheld infrared temperature sensor. This difference was within the reported error for the camera (±3°C) and infrared sensor (±2°C). This margin of error is comparable to that of other FLIR imaging calibration experiments (Barbieri et al., 2010).

During each data collection session with the dolphins housed in an outdoor pool, we recorded air temperature (mean±s.d.; 18.1±3.80°C), water temperature (19.8±2.19°C), humidity (69.8±15.9%), cloud level (474.57±675.44 m), solar radiation (326±245 W m^−2^) and wind speed (22.56±13.34 km h^−1^). Only ambient temperature (18.74±0.55°C), water temperature (15.08±0.13°C) and humidity (49.41±16.28%) were recorded for the belugas in the indoor facility. Ambient temperature and humidity were changed daily in the camera settings before image collection. The reflected temperature was kept at 20°C, and emissivity was 0.98. Images were always taken 1 m above the fluke, and the lens was kept parallel to the fluke to keep the angle of incidence close to 0 deg.

Analysis of the thermal images of the ventral fluke surface was conducted using FLIR Thermal Studio software (Teledyne FLIR). Five horizontal transects were drawn to determine regional average and maximum surface temperatures. These values were used to estimate surface heat patterns. To determine changes in regional thermal patterns associated with vasodilation, the proportion of fluke area (pixels) exceeding a temperature threshold was quantified using ImageJ (National Institutes of Health, Bethesda, MD, USA). Note that this proxy offers insights into relative surface warming, it does not directly quantify blood perfusion, which is also influenced by environmental cooling, convective heat loss and tissue insulation.

### Analysis and statistics

We defined ‘exercise recovery’ to occur after mean values of each metric were greater than one standard deviation from average resting values (Table S1). We defined ‘return to rest’ as values that were less than one standard deviation of average resting values after the defined period of exercise recovery. In cases when a return to rest was not identifiable (i.e. the metric did not return to rest in the observed recovery period), predicted rates of change were calculated as an alternate metric to surface recovery behaviors. For all models, residuals were visually inspected for normality and homogeneity of variance. Diagnostic plots were also used to select the distribution family for each model. All analyses were completed in R (v4.4.1; https://www.r-project.org/) using packages from the tidyverse suite (Wickham et al., 2019). Outputs from species-specific models and summary statistics were used to compare the recovery timelines, magnitude of change away from rest, and the rate of change during the surface recovery period for each metric at the species level.

### Metabolic gas recovery timelines

Subsampled oxygen and carbon dioxide content were corrected for standard temperature and pressure and converted to *V̇*_O_2_ _and *V̇*_CO_2__ using eqns 11.7 and 11.8, respectively, from Lighton (2018). Values are reported as ml O_2_ kg^−1^ min^−1^ or ml CO_2_ kg^−1^ min^−1^ depending on the respired gas. For resting measurements, we recorded the lowest mean oxygen consumption or carbon dioxide production for at least 5 min per trial, allowing animals to reach a steady state (John et al., 2024). Submerged sequential swimming and SAB metabolic rates were measured after the final swim sequence or behavior (Fig. 2). Exercising (swims, SABs) metabolic rates were measured as the difference between post-exercise *V̇*_O_2_ _and *V̇*_CO_2__ values during recovery and resting (John et al., 2024; Williams et al., 2017a). We also recorded the recovery duration, which was the elapsed time for *V̇*_O_2_ _and *V̇*_CO_2__ values to return to rest. Lastly, we calculated the respiratory exchange ratio (RER) by dividing the mean *V̇*_CO_2__ by the mean *V̇*_O_2__ for both the exercising and resting phases of each trial.

A generalized linear mixed-effect model (GLMM) framework with the glmmTMB package (Brooks et al., 2017) was used to infer the effect of gas type (O_2_, CO_2_) on recovery durations following exercise. The response variable was recovery duration in separate models for each species. Interactions between trial type (i.e. repetitive swims, SABs) and gas type were included as fixed effects to account for the trial-specific effects of gas on recovery times. Animal ID was included as a random effect to account for the variation associated with each animal and repeated measures. A gamma distribution with a log link function was used. To report coefficient predictions, 1000 simulations of the model coefficients were generated using the ‘mvnorm’ function from the MASS package (v7.3-61; Venables and Ripley, 2002). A likelihood ratio test was also used to compare models that did and did not include gas type as a covariate. Lastly, to compare species effects on respiratory recovery times, a GLMM was developed with trial type, gas type and species included as fixed effects, and animal ID as a random effect. Interaction terms were included for trial and gas type, trial and species, and gas type and species. A gamma distribution was used with a log link function.

The means and standard deviations for each blood gas metric were recorded according to species and trial type. Blood samples were grouped into 1 min bins before summary statistics were calculated. If a standard deviation is not presented, it indicates that only one sample was taken for that 1 min bin. Generalized linear model (GLM) frameworks were used to investigate how blood *P*_O_2__, *P*_CO_2__, lactate and HCO_3_^−^ changed with time at the surface for each species. For the GLMs, an interaction between trial type and sampling time, animal ID and date were included as fixed effects. A gamma distribution with a log link function was selected. Separate models were developed for *P*_O_2_ _and *P*_CO_2__. We quantified uncertainty in blood gas levels at two time points (immediately following exercise and the mean change over 10 min) via parametric bootstrapping of the confidence interval. For each blood gas model, we sampled the model coefficients 1000 times from a multivariate normal distribution defined by the model's coefficient estimates and variance–covariance matrix. Then we predicted the response blood gas metric, using the sampled coefficients, at time points 0 and 10 min. Although beluga measurements were available out to 15 min, we generated predictions for 10 min to better compare across species.

GLMMs were used to model the effect of *P*_CO_2__ and lactate on blood pH, and separate models were developed for each species. Trial type and an interaction between trial type and *P*_CO_2__ and/or lactate were included as fixed effects, and date and animal ID were included as random effects. A Gaussian distribution was used with a log link function.

Additional GLMs were developed for each species to investigate how blood pH changed with time. Trial type, sampling time, animal ID and date were included as fixed effects. A gamma distribution with a log link function was used. Separate models were developed for each species. The remainder of the analyses were identical to those described above for blood gases.

### Integrative recovery timelines: systems transporting metabolic gases

Sufficient data were available to identify the return to rest for both breathing pattern metrics (breath duration and IBI). Breath frequency (breaths min^−1^) was also determined by taking the inverse of the IBI (seconds between breaths). Asymptotic regression models were used to assess the impact of trial type on each breathing pattern metric given their anticipated asymptotic relationships with time (*t*) (Eqn 1):
(1)

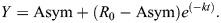


Separate models were developed for each species. For each breathing pattern model, the rate constant (*k*) varied with trial type, whereas the asymptote (Asym) and *y*-intercept (*R*_0_) were fixed. We also accounted for temporal autocorrelation grouped by animal ID and date. If the effect of trial type was significant, a *post hoc* Tukey test was performed for pairwise comparisons with a Bonferroni significance adjustment using the ‘emmeans’ function from the emmeans package (https://CRAN.R-project.org/package=emmeans).

Sufficient data were also available to identify the return to rest for the vasculature metrics (mean skin surface temperature, maximum surface temperature and percent perfusion). Separate GLMMs were used per species to assess the influence of trial type on the mean and maximum skin surface temperature models. Here, a gamma distribution with a log link function was selected. The percent perfusion data were zero-inflated and ranged from 0 to 1, thus a GLMM with a beta distribution and logit link function was developed. We allowed zero inflation to vary by trial type. For all vasculature models, animal ID and an interaction between time and trial type were included as covariates. Lastly, we accounted for temporal autocorrelation grouped by date and animal ID.

## RESULTS

### Respiratory gases

**Fig. 3.**
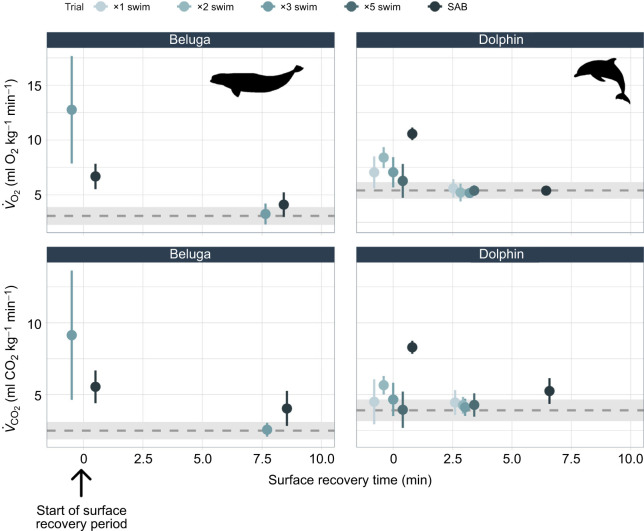
**Respiratory gas recovery timelines.** Mean±1 s.d. of oxygen consumption (*V̇*_O_2__) and carbon dioxide production rates (*V̇*_CO_2__) in relation to recovery times. Points are separated by species and trial type [swim repetitions, surface-active behaviors (SABs)]. Respiratory gas measurements were taken at the start of the surface recovery period (means plotted near 0 min) and when the animal returned to resting values (determined during data collection). Species-specific mean±1 s.d. of resting respiratory gases are plotted with the grey dashed line and rectangles, respectively.

Recovery time at the water's surface for *V̇*_O_2_ _and *V̇*_CO_2__ did not significantly differ by metabolic gas type (O_2_, CO_2_) for either species. Results from the likelihood ratio test also suggested that a respiratory gas recovery time model considering gas type was not significantly different from one that omitted gas type as a predictor variable. Recovery times were positively correlated with swim count, with SABs having the longest surface recovery times for both gases and species (Fig. 3; Table S2). For dolphins, ×5 swims and SABs had significantly longer recovery times than ×1 swims (*P*_×5swim_=0.00058; *P*_SAB_=2×10^−16^). Additionally, *V̇*_CO_2__ did not return to average resting values following SABs throughout the study period, whereas *V̇*_O_2_ _did for both species. For SABs, mean RER values during the resting phase were 0.98 for belugas and 0.98 for dolphins, indicating that CO_2_ was not fully recovered (Table S2). Species type significantly influenced surface recovery times of metabolic rates (*V̇*_O_2_ _and *V̇*_CO_2__; *P=*2×10^−16^), where beluga whale recovery times were 2.51±1.11 times longer than those for dolphins (averaged across gases and exercise types).

### Blood gases, lactate and pH

**Fig. 4.**
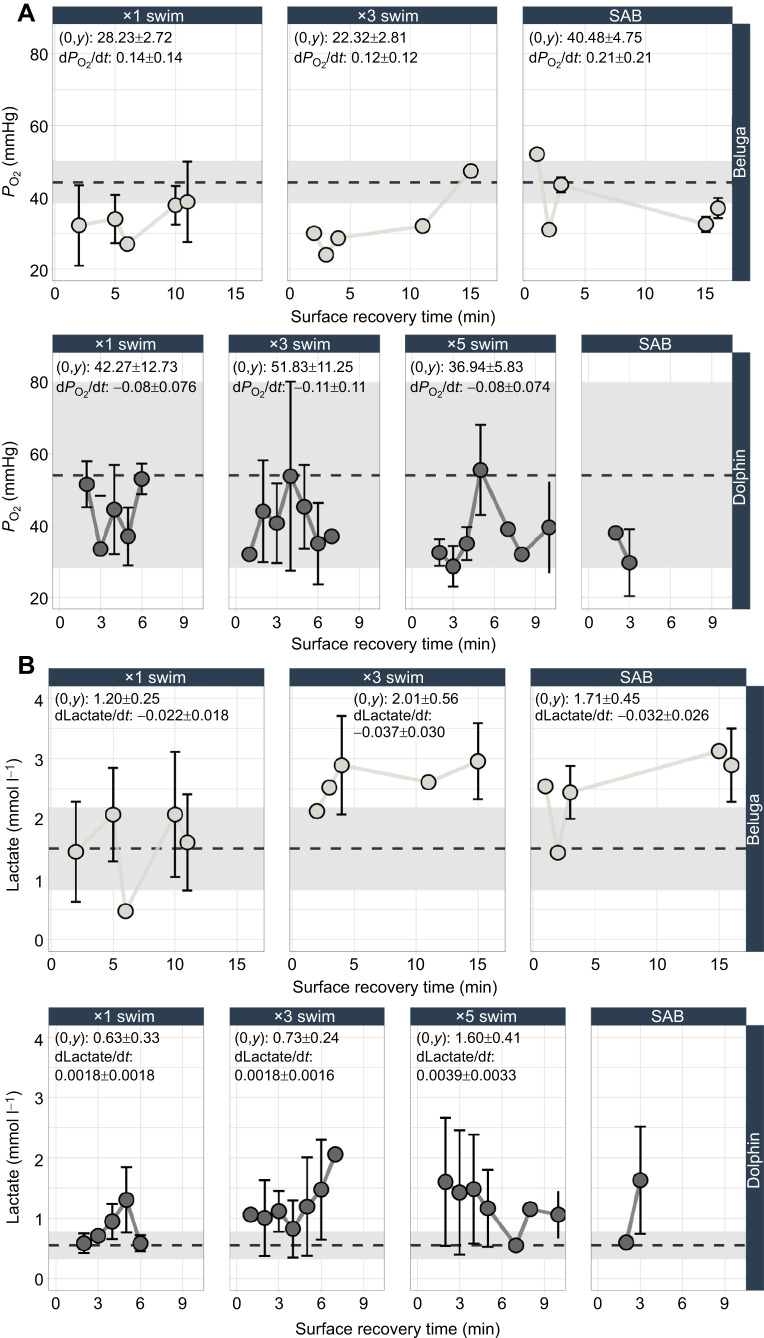
**Blood O_2_ and lactate recovery timelines.** Mean (A) oxygen (*P*_O_2__) and (B) lactate for belugas and bottlenose dolphins. Points represent the mean grouped by 1 min bins and averaged across animals. Error bars (±1 s.d.) for each 1 min bin are presented when multiple points were taken for a given bin. Species-specific means±1 s.d. of resting blood gases are plotted with the grey dashed line and rectangles, respectively. The modeled *y*-intercept (0,*y*) and rate of change were calculated from 0 to 10 min for both species, and are presented for each trial–species combination. The *y*-intercept and rate of change are not presented for dolphin SABs as these data were only collected at ∼3 min.

Larger changes in *P*_O_2__ were observed in belugas compared with dolphins, whereas lactate concentrations remained elevated from resting values for both species throughout the observed study period. Maximal swim counts resulted in the greatest changes in *P*_O_2__ from resting values for each species (beluga resting *P*_O_2__: 44.170±5.640 mmHg; dolphin resting *P*_O_2__: 54±25.65 mmHg) as identified by the modeled *y*-intercepts (Fig. 4A; Table S3). Across trials, beluga whales showed an initial reduction in *P*_O_2_ _values below resting values and recovered within 12–16 min depending on the trial. Additionally, beluga whales had the largest reductions in *P*_O_2__ during the ×3 swim trials relative to all other exercise levels. The *P*_O_2__ of dolphins generally remained within resting values across the observed surface recovery period (7–10 min). Large, prolonged changes in lactate were observed for both species, with the largest changes (as indicated by the modeled *y*-intercepts) occurring after maximal swim repetitions (Fig. 4B; Table S3). For belugas, lactate remained above resting levels following both ×3 swims (1.8 times resting) and SABs (1.6 times resting) throughout the observed recovery period (16 min). For dolphins, lactate also remained elevated throughout the observed recovery period (7–10 min) following ×3 swims (2 times resting), ×5 swims (2.3 times resting) and SABs (2.5 times resting). The fastest rate of change back to resting levels for belugas followed ×3 swims. Conversely, recovery rate did not change across swim repetitions for dolphins.

**Fig. 5.**
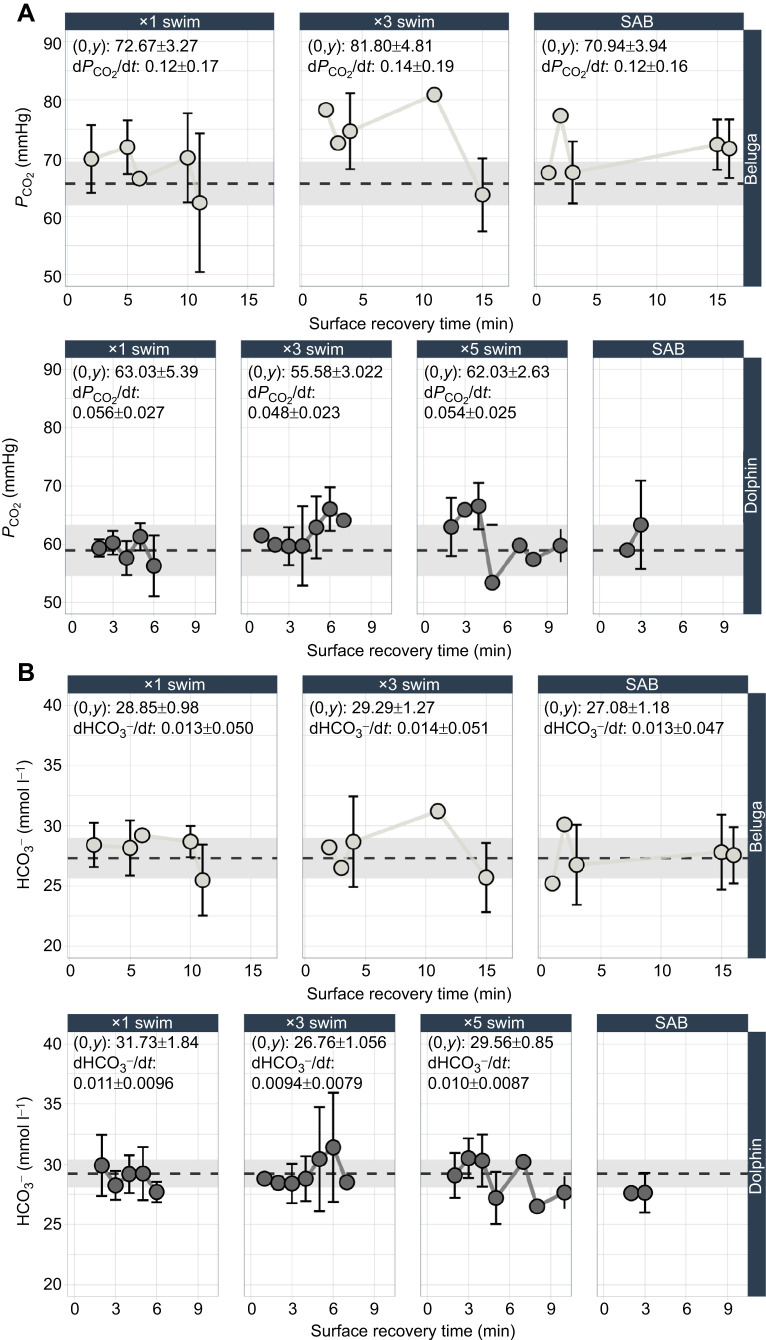
**Blood CO_2_ and HCO_3_^−^ recovery timelines.** Mean (A) carbon dioxide (*P*_CO_2__) and (B) bicarbonate (HCO_3_^−^) for belugas and bottlenose dolphins. Points represent the mean grouped by 1 min bins and averaged across animals. Error bars (±1 s.d.) for each 1 min bin are presented when multiple points were taken for a given bin. Species-specific means±1 s.d. of resting blood gases are plotted with the grey dashed line and rectangles, respectively. The modeled *y*-intercept (0,*y*) and rate of change were calculated from 0 to 10 min for both species, and are presented for each trial–species combination. The *y*-intercept and rate of change are not presented for dolphin surface-active behaviors (SABs) as these data were only collected at ∼3 min.

Similar to *P*_O_2__, larger changes in *P*_CO_2__ were observed in belugas compared with dolphins, whereas only minor changes in associated HCO_3_^−^ were seen for both species. Maximal swim counts resulted in larger changes in *P*_CO_2__ relative to resting values for each species (beluga resting *P*_CO_2__: 65.65±3.61 mmHg; dolphin resting *P*_CO_2__=58.95±4.32 mmHg) as identified by the modeled *y*-intercepts (Fig. 5A; Table S3). The *P*_CO_2__ of beluga whales also remained within or returned to resting values during the post-exercise recovery period, following similar recovery timelines as *P*_O_2__. The fastest rate of change for beluga *P*_CO_2__ values (d*P*_CO_2__/d*t*) also occurred during maximal swim counts. Dolphin *P*_CO_2__ values departed further from rest relative to *P*_O_2__ values during the ×3 and ×5 swim trials. For this odontocete, *P*_CO_2__ remained elevated at the end of the study period (7 min) or returned to rest (5 min) depending on the level of exercise. *y*-intercepts and rates of change are not presented for dolphin SABs, as these samples were only collected for a single time period. Overall, belugas had faster rates of change back to resting values, whereas dolphins showed smaller changes from resting values and faster returns to resting levels. For both species, HCO_3_^−^ remained within resting levels across trial types (Fig. 5B; Table S3).

**Fig. 6.**
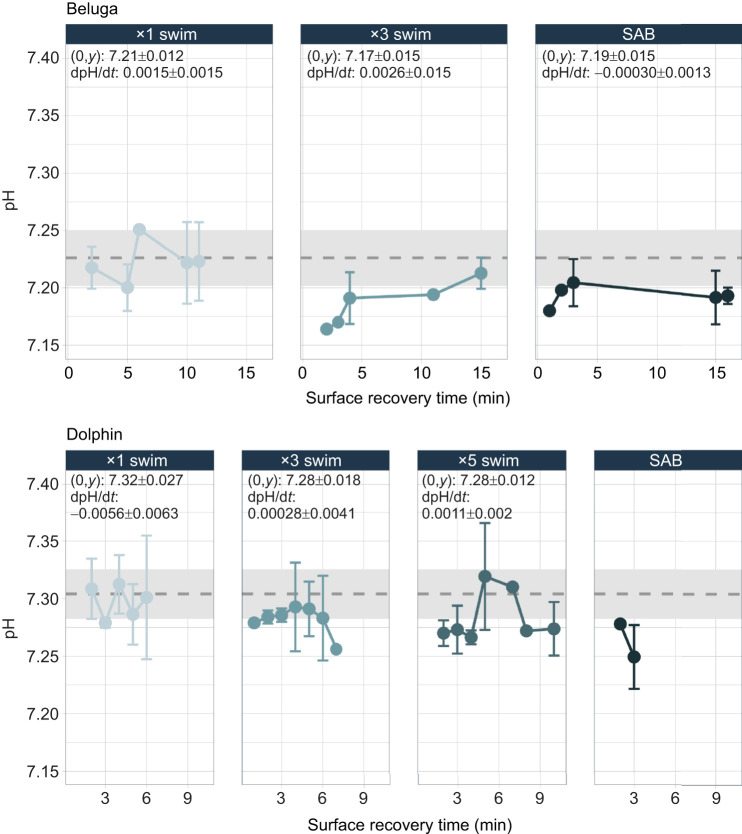
**Blood pH recovery timelines.** Mean blood pH for belugas and bottlenose dolphins. Less transparent points represent the mean grouped by 1 min bins and averaged across animals. Error bars (±1 s.d.) for each 1 min bin are presented when multiple points were taken for a given bin. Species-specific means±1 s.d. of resting blood pH are plotted with the grey dashed line and rectangles, respectively. The modeled *y*-intercept (0,*y*) and rate of change were calculated from 0 to 10 min for both species, and are presented for each trial–species combination. The *y*-intercept and rate of change are not presented for dolphin SABs as these data were only collected at ∼3 min.

For both species, models for blood pH considering *P*_CO_2__ (belugas: BIC=−201, RMSE=0.008; dolphins: BIC=−273.4, RMSE=0.011) outperformed those with lactate (belugas: BIC=−175.7, RMSE=0.012; dolphins: BIC=−228, RMSE=0.021). Exploratory pH models that included both *P*_CO_2__ and lactate performed similarly to the models that considered *P*_CO_2__ alone for both species (belugas: BIC=−200.1, RMSE=0.005; dolphins: BIC=−260.9, RMSE =0.010). Following the highest swim counts (×3), belugas showed larger changes in blood pH from resting values compared with dolphins. The larger odontocetes also had the fastest rate of change (dpH/d*t*) back to resting values (0.0026±0.0014) (Fig. 6). Following the ×3 swims, belugas returned to rest at approximately 16 min but stayed below resting values following SABs for the entire observation period (16 min). Blood pH of dolphins declined with exercise intensity, with the lowest modeled *y*-intercept following the ×5 swim trials. Across trial types, dolphin dpH/d*t* was low, and did not return to resting values during the observation period (6–10 min). For both species, blood pH declined with exercise intensity, with belugas showing larger changes in blood pH from rest compared with dolphins, and faster rates of change back to rest following the same swim repetitions. Blood acidification following increasing exercise intensity is likely attributed to both metabolic and respiratory acidosis (Fig. S3). Some species-specific differences in blood acidification can be observed, where declining blood pH may be more associated with metabolic acidosis (i.e. declines in HCO_3_^−^) for dolphins, and respiratory acidosis (i.e. accumulating CO_2_) for belugas.

### Respiratory and vascular gas transport systems

**Fig. 7.**
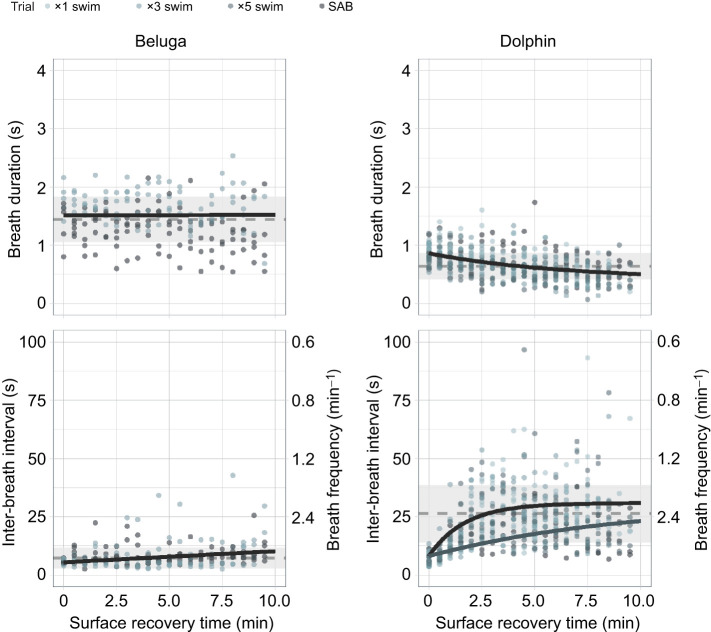
**Breathing pattern recovery timelines.** Breath durations and inter-breath intervals plotted by species and trial type (swim repetitions, SABs). Points represent the observed data color-coded by trial type. Asymptotic model predictions were averaged across trial types that were not significantly different (black) or were plotted separately if there were significant differences (*P<*0.05). The only instance where a significant difference was observed was for dolphin IBI between SABs (blue line) and the sequential swims (black line). Species-specific means±1 s.d. of resting breathing pattern parameters are plotted with the grey dashed line and rectangles, respectively.

For beluga whales, trial type had no significant effect on the rate of change back to rest for both breathing pattern metrics, which remained within resting values throughout the observation period (10 min) (Fig. 7; Table S4). In comparison, trial type did not significantly affect the rate of recovery for breath duration in dolphins. However, following SABs, the rate of return for the IBI was significantly slower than following the ×1 swims in dolphins (*P*=0.010, *t*_IBI_=∼2.5 min delayed recovery).

**Fig. 8.**
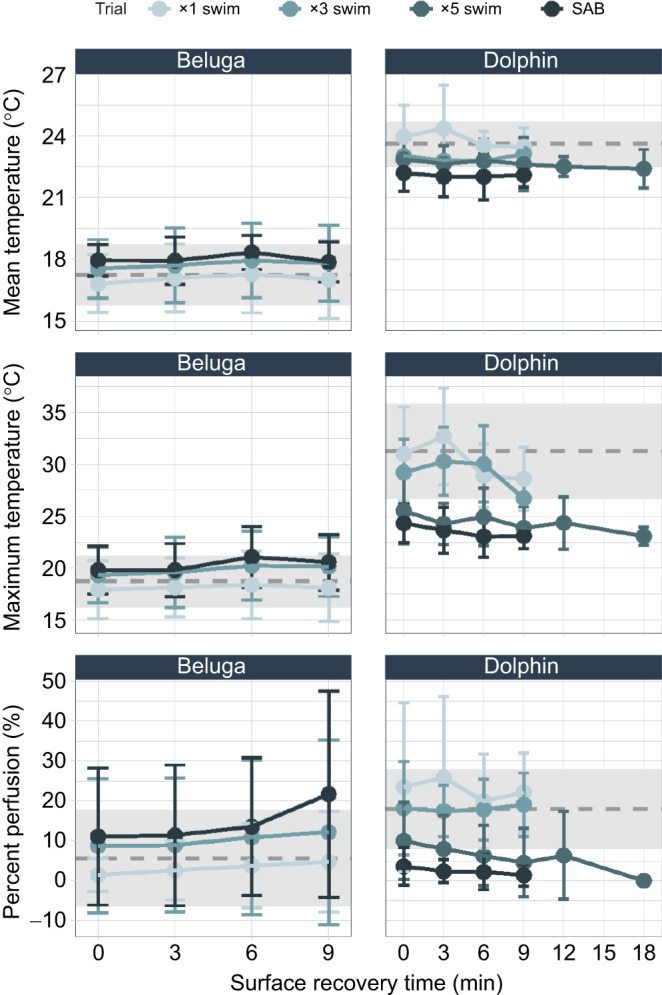
**Fluke vasculature recovery timelines.** Points represent the mean of each metric per 3 min bin averaged across animals. Error bars (±1 s.d.) for each 3 min bin are also presented. Species-specific means±1 s.d. of resting are plotted with the grey dashed line and rectangles, respectively. Points are separated by species and trial type (swim repetitions, SABs).

Trial type had no significant effect on any of the vasculature metrics for beluga whales. Mean and maximum skin surface temperatures also remained within resting values throughout the observation period (9 min; Fig. 8; Table S5). Percent area where vasodilation was observed (i.e. percent local perfusion) remained within resting values for the ×1 and ×3 swims. In contrast, the dilated area increased throughout the observation period following SABs but was not significant at the 0.05 level (*P*=0.062). The response differed for dolphins. Trial type significantly influenced each of the vasculature metrics for dolphins. Mean skin surface temperatures were significantly cooler (Δ=1.95°C) following SABs (*P*=0.049) compared with the ×1 swims. Maximum skin surface temperatures were significantly colder as exercise intensity increased; this was more evident for ×5 swims and SABs than the other exercise types (*P*<0.05; note that the difference between ×5 swims and SABs was not significant). Less vasodilation as monitored by percent perfusion occurred during recovery from more intense exercise, where SABs showed the smallest area relative to all other trial types (*P*_×5swim–SAB_=0.0015). These differences in skin temperature and percent perfusion persisted throughout the observation period (9–18 min depending on the exercise type). Vasculature metrics during recovery from ×1 and ×3 swims remained within resting values throughout the observation period (9 min). These metrics remained below resting values throughout the recovery observation period following ×5 swims and SABs (×5 swims: 18 min; SABs: 9 min).

## DISCUSSION

Recovery during inter-dive surface intervals is a critical component of sequential diving by marine mammals. Indeed, much of the physiology and behavior of diving mammals is centered on minimizing time readjusting metabolic gases and extending time at depth. This physiological optimization provides many positive ecological fitness effects, including maximizing foraging efficiency, and reduces periods with elevated predation risk or exposure to human–wildlife conflicts (Beltran et al., 2021; Davis, 2014; Erbe et al., 2019). Consequently, physiological mechanisms that influence surface recovery durations (e.g. oxygen replenishment and carbon dioxide offloading) will affect the partitioning of time that divers spend at depth or on the surface. Our results indicate a cascade of recovery events along the oxygen pathway for odontocetes that is influenced by exercise level and species. In general, dolphins and beluga whales show similar recovery times for their individual respiratory and blood O_2_ and CO_2_ levels. Lactate accumulation and the accompanying acidification of blood pH, best explained by CO_2_ accumulation, were among the slowest metrics to recover following repetitive submerged swimming for the odontocetes in this study. Despite these similarities for dolphins and belugas, our results also indicate distinct, species-specific differences in breathing patterns and peripheral perfusion in response to repetitive swims. Together, these results imply a significant role for CO_2_ readjustments in shaping overall recovery timelines.

### O_2_ and CO_2_ recovery

Previous studies have reported that the restoration of blood *P*_CO_2__ following diving requires comparatively longer recovery periods than *P*_O_2__ owing to the intermediate phases of CO_2_ storage (HCO_3_^−^) (Boutilier et al., 2001). In addition, CO_2_ recovery may require more breaths than O_2_ for recovery, necessitating increased surface durations to return to resting levels (Gerlinsky et al., 2014; Rosen et al., 2017). Instead, we found that the observed changes in respiratory O_2_ and CO_2_ levels resulted in only minor extensions in metabolic gas recovery times (<1 min) with increased swim repetitions by dolphins and beluga whales (Fig. 3). Although a possible mechanism for this response may be associated with lowered metabolic rates associated with diving (Sparling and Fedak, 2004), it is unlikely for the swimming exercise examined here. Extended recovery durations were required by the odontocetes in the present study for both gases following a punctuated ‘burst’ of high energy exercise during SABs. This was especially evident for respiratory *V̇*_CO_2__, which did not completely return to rest whereas *V̇*_O_2__ did.

Blood gases in beluga whales recovered within similar durations across exercise types. Conversely, dolphin *P*_O_2__ values remained within or near resting values, whereas *P*_CO_2__ was elevated throughout the observation period following moderate swimming repetitions (Fig. 4A; Fig. 5A). The recovery timelines observed for dolphins more closely align with what is reported in previous studies (Boutilier et al., 2001; Gerlinsky et al., 2014), reinforcing the unique recovery patterns for blood O_2_ and CO_2_ in belugas. This larger, longer diving odontocete also demonstrated larger *P*_CO_2__ accumulation at the start of the surface recovery period compared with the dolphins, although the values were within range of other studies investigating *P*_CO_2__ accumulation in belugas during submerged swimming (Shaffer et al., 1997). Previous work has also reported comparatively higher skeletal muscle buffering capacities in beluga whales, which may support their ability to rapidly recover from these relatively larger fluctuations in blood CO_2_ (Noren, 2004).

### Blood pH recovery

Previous studies (i.e. Boutilier et al., 1993; Castellini and Somero, 1981; Lenfant et al., 1970) have suggested that marine mammals have higher blood pH buffering capacities relative to terrestrial mammals of a similar size, resulting in a higher tolerance to lactate and CO_2_ accumulation at depth. However, note that blood gases, changes in lactate, and blood pH do not always show predictive relationship patterns. For example, Noren et al. (2012b) and Stockard et al. (2007) reported minimal changes in lactate levels in association with large changes in respiratory gases and blood pH. In the present study, as exercise intensity increased, we also saw the blood pH become more acidic and lactate begin to accumulate (Fig. 6; Table S3). Despite lactate remaining elevated, *P*_CO_2__ was a better predictor for blood pH, indicating the influential role for *P*_CO_2__ in driving blood pH of odontocetes performing moderate to high levels of exercise examined as here. Given the duration of our observation period (10 min for dolphins and 15 min for belugas), this relationship could be partially driven by the different rates that protons released during exercise and accumulating lactate leave the muscle (Bangsbo et al., 1997).

Blood buffering capacities of marine mammals are expected to vary with body size and swimming or diving ability, with species that routinely complete burst speed swimming or have exceptionally long dive durations having the greatest buffering capacities (Noren, 2004; Noren et al., 2012b). The recovery of blood pH lagged behind respiratory gases for both species (∼10 min or never fully returning to rest) (Fig. 6). Although belugas showed larger declines in blood pH following maximal swim repetitions compared with dolphins, they also displayed faster rates of change back to resting values. This could possibly be attributed to their increased capacity to buffer acidic metabolic byproducts, enabling them to limit more dramatic changes in blood pH. The faster rates of change compared with dolphins may also be supported by the differences observed for the processes supporting CO_2_ transport out of the system (i.e. increased peripheral vasodilation with activity, more frequent and longer breaths) (Figs 7, 8). The slow recovery of blood pH and lactate relative to blood *P*_O_2__ and *P*_CO_2__ indicates that they may serve as a more influential chemosensory trigger, potentially extending system-wide recovery timelines and serving as the overarching limitation to odontocete dive recovery.

### Metabolic gas transport system recovery

Generally, the buildup of CO_2_ drives changes in breathing patterns among odontocetes and other marine mammals (Blix, 2018; Boutilier et al., 2001; Butler, 1982; Gerlinsky, 2013; Rosen et al., 2017). Here, we only observed small changes in breath duration and IBIs of beluga whales despite marked changes in onboard respiratory gases and blood acidification (Fig. 7; Table S4). For bottlenose dolphins, we recorded changes in IBI recovery timelines following SABs, with a delayed return to resting levels following these ‘burst’ activities. Little to no changes in breathing patterns were observed with increasing swim repetitions. Both *P*_CO_2__ and blood pH can serve as ventilatory cues for marine mammals, with the latter typically requiring longer recovery durations than indicated by ventilatory recovery timelines (Kooyman et al., 1973; Noren et al., 2012b). This temporal mismatch in surface recovery requirements and physiological homeostasis is important to note, as ventilatory behavior is often used as a proxy for dive cost and recovery in free-ranging odontocetes (Christiansen et al., 2014; Isojunno et al., 2018; Williams et al., 2022). Because breathing rates do not necessarily reflect system-wide recovery, merely monitoring breathing patterns may not accurately indicate the total response and recovery of free-ranging individuals.

Peripheral vasoconstriction is a pillar of the mammalian dive response, and is maintained by neurotransmitters during apnea (Blix, 2018; Gooden and Elsner, 1985; McArdle et al., 1957; Thornton and Hochachka, 2004). As the diver returns to the surface to breathe, it is expected that vasodilation of the skeletal muscles resumes, concomitant with increased cardiac output and ventilation rates, to enable metabolic by-product removal and metabolic gas recovery (Malte et al., 2016; Meir and Ponganis, 2010). However, the opposing effects of nervous-system-mediated vasoconstriction and vasodilation caused by accumulated metabolic by-products can lead to varying perfusion outcomes (Gooden and Elsner, 1985). In the present study, beluga whales and bottlenose dolphins had opposing post-exercise vascular responses upon surfacing (Fig. 8; Table S5). Mean and maximum skin surface temperatures of beluga whales across the fluke generally remained within resting values across trial types, potentially indicating a muted response to the changing blood gases, lactate and pH. However, as exercise intensity increased, the percent area with observed vasodilation (i.e. percent perfusion) increased throughout the surface recovery period, exceeding resting values following SABs. This result is in agreement with past research documenting peripheral reperfusion following exercise, which would support rapid O_2_ replenishment and CO_2_ offloading throughout the system (Favilla et al., 2022; McDonald and Ponganis, 2013; Meir and Ponganis, 2010). In contrast, vasodilation of the dolphin flukes showed a negative relationship with exercise intensity, where vasodilation of the fluke was significantly reduced following the most intense behaviors. Likely, the prolonged vasoconstriction in the extremities observed for dolphins will delay the exposure of sensitive organs (particularly the heart, lungs and brain) to persistent blood acidification. For humans, it has been suggested that extended blood acidification may prevent cell and tissue damage due to reperfusion injury (i.e. exposure to oxygen free radicals) (Caldwell-Kenkel et al., 1995; Cohen et al., 2007; Ponganis, 2015). Given that marine mammals regularly experience extreme prolonged vasoconstriction, this potential function of delayed perfusion and resulting local acidification deserves further investigation. For both odontocetes in the present study, changes in peripheral vasodilation persisted for 9 or 18 min depending on exercise intensity, making it one of the slowest metrics to recover.

### Species level differences

Species responses are likely shaped by the complicated interaction between body mass, species-specific diving adaptations and preferred diving patterns of wild counterparts (i.e. typical swim speeds, dive durations and sprint speeds). Larger body sizes observed for beluga whales compared with bottlenose dolphins would result in lower metabolic rates, facilitating increased aerobic dive capacities and reduced lactate accumulation (Noren et al., 2012b). It has also been documented that beluga whales have higher myoglobin concentrations and muscle buffering capacities relative to bottlenose dolphins, which would significantly extend their diving ability and increase their recovery processes (Noren, 2004; Noren and Williams, 2000). Diving ability and recovery can also be influenced by diving patterns, where the longest diving or fastest swimming cetaceans tend to have the highest buffering capacities counteracting CO_2_ and lactate accumulation or blood acidification (Noren, 2004). Thus, the complex and interactive effects between body size, species-specific diving adaptations and diving patterns collectively influence the diving ability and recovery processes observed in this study. Further investigation is needed to better understand the relationships between these various factors, how they each influence dive recovery requirements, and how their combined effects vary across species.

### Implications and limitations

Understanding recovery from perturbations in metabolic gas management is especially relevant for coastal and deep-diving species that may regularly experience a delay in blood pH recovery. The high energy SABs in this study were included to simulate some of the avoidance behaviors observed in wild odontocete populations following human disturbance (e.g. vessel presence, acoustic emissions) (Noren et al., 2012a). These behaviors may be more difficult for the animal to sustain compared with the swim repetitions, which are supported through graded metabolic readjustments associated with the dive response (Elmegaard et al., 2016; McDonald and Ponganis, 2014). Thus, such high-intensity surface behaviors in the wild may result in larger changes in metabolic gases and extended recovery requirements for impacted animals. The effect of SABs was conserved across species, although dolphin recovery times showed greater extensions compared with beluga whales.

Limitations of this study include a small sample size and the controlled environment with trained animals. Consequently, we did not account for the many other complex factors (e.g. hormonal changes, faster swimming speeds, longer dive durations) that may affect the recovery of freely diving wild odontocetes following the range of natural behaviors that may trigger CO_2_ accumulation (e.g. flight response due to human disturbance, foraging opportunities). Additionally, we collected data from dolphins and beluga whales following different exercise durations and using different sampling regimes to balance multiple animal performance preferences and varied recovery processes. Although our approaches enabled us to document whole-animal recovery following graded exercise, it did constrain our ability to make specific ‘swim to swim’ comparisons, which is an important next step for follow-up work. In view of this, future work should prioritize investigating surface recovery for more species of different masses and diving abilities, include additional physiological systems involved in maintaining homeostasis of metabolic gases (e.g. heart rate, cortisol levels), and include additional behaviors observed in free-ranging individuals (e.g. swims simulating foraging dives, speed swimming).

Across physiological metrics and for both species examined here, blood pH, lactate and peripheral perfusion had the longest recovery timelines and were not observed to return to average resting values throughout the surface observation period. Previous work has suggested that CO_2_ accumulation and depletion may determine surface recovery durations, rather than O_2_ (Boutilier et al., 2001; Gerlinsky et al., 2014; Rosen et al., 2017). The results of the present study demonstrated a different species-specific pattern. Rather, we found that blood acidification caused by the compounding effects of CO_2_ and lactate accumulation, and their influence on peripheral perfusion, may be among the slowest physiological processes to recover from sequential swimming. These data provide a new understanding of complicated physiological relationships and potential physiological limitations of the post-submergence recovery period of odontocetes following sequential swimming with minimal recovery opportunities.

## Supplementary Material

10.1242/jexbio.251853_sup1 Supplementary information
