# Supplementary material for: Effects of carbon dioxide accumulation on post-dive physiological recovery in odontocetes
Source: J Exp Biol. 2026 Apr 13;229(7):jeb251853. doi: 10.1242/jeb.251853 (PMC13120684; doi:10.1242/jeb.251853)
Supplement: Supplementary information [file jexbio-229-251853-s1.pdf]

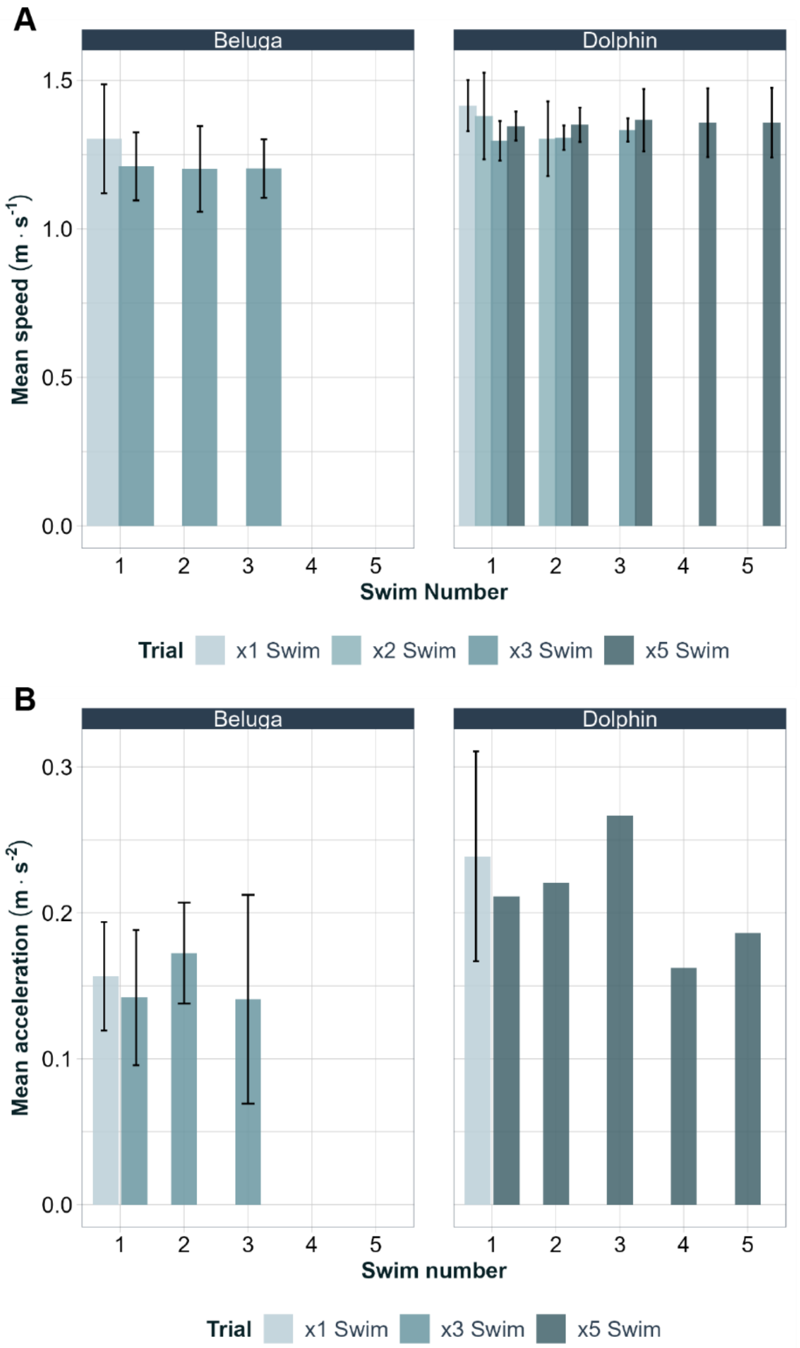

Fig. S1. Average A) speed and B) accelerometry across swim repetitions (swim number) and species. Error bars represent 1 s.d. away from the mean. No error bars are present for x5 swims for dolphins as this was only completed one time for one animal.

Journal of Experimental Biology • Supplementary information

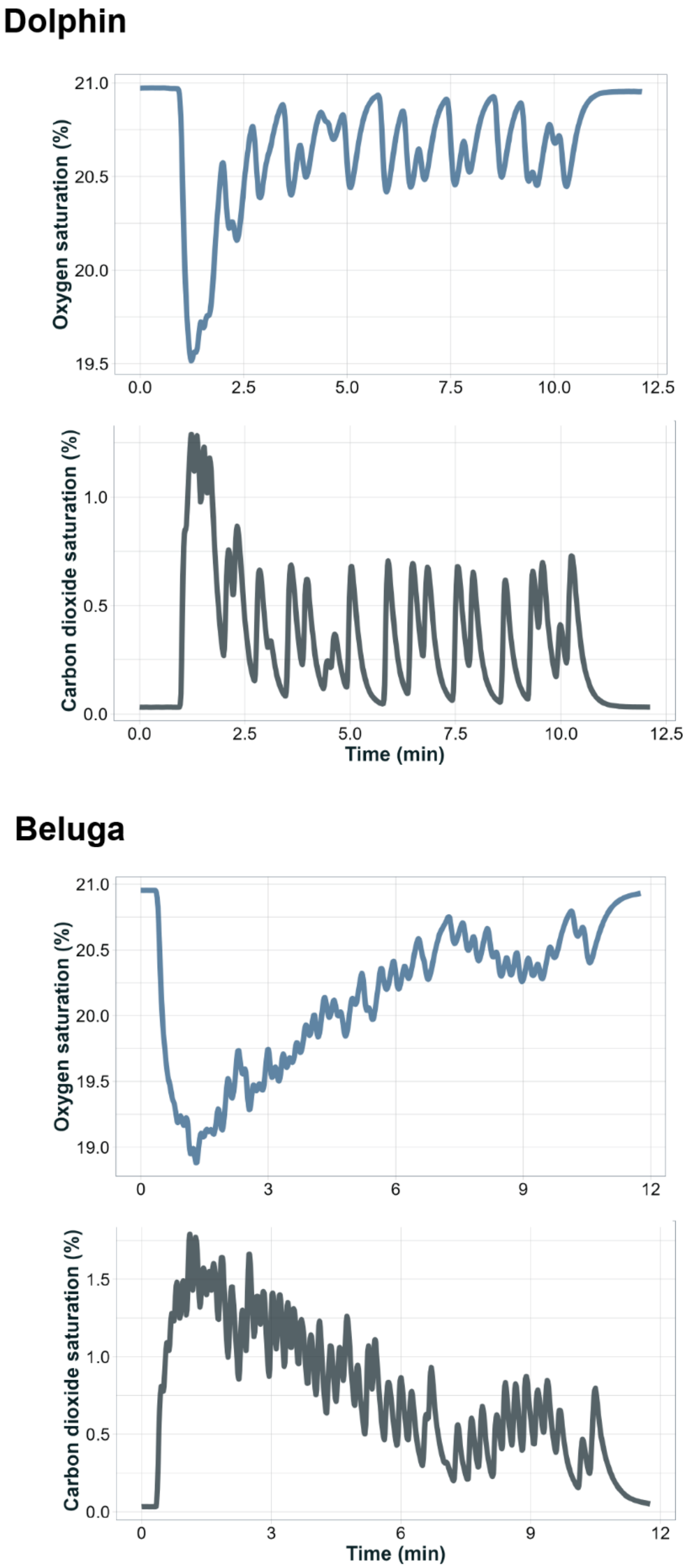

Fig. S2. Oxygen and carbon dioxide saturation percentages within the metabolic dome for dolphins (top) and beluga whales (bottom). Saturation percentages are from a representative trial for each species (dolphin: x5 swim; beluga: x3 swim) for one animal, but represent a typical swim recovery pattern (initial drop or increase depending on the gas followed by a tapering off period).

Journal of Experimental Biology • Supplementary information

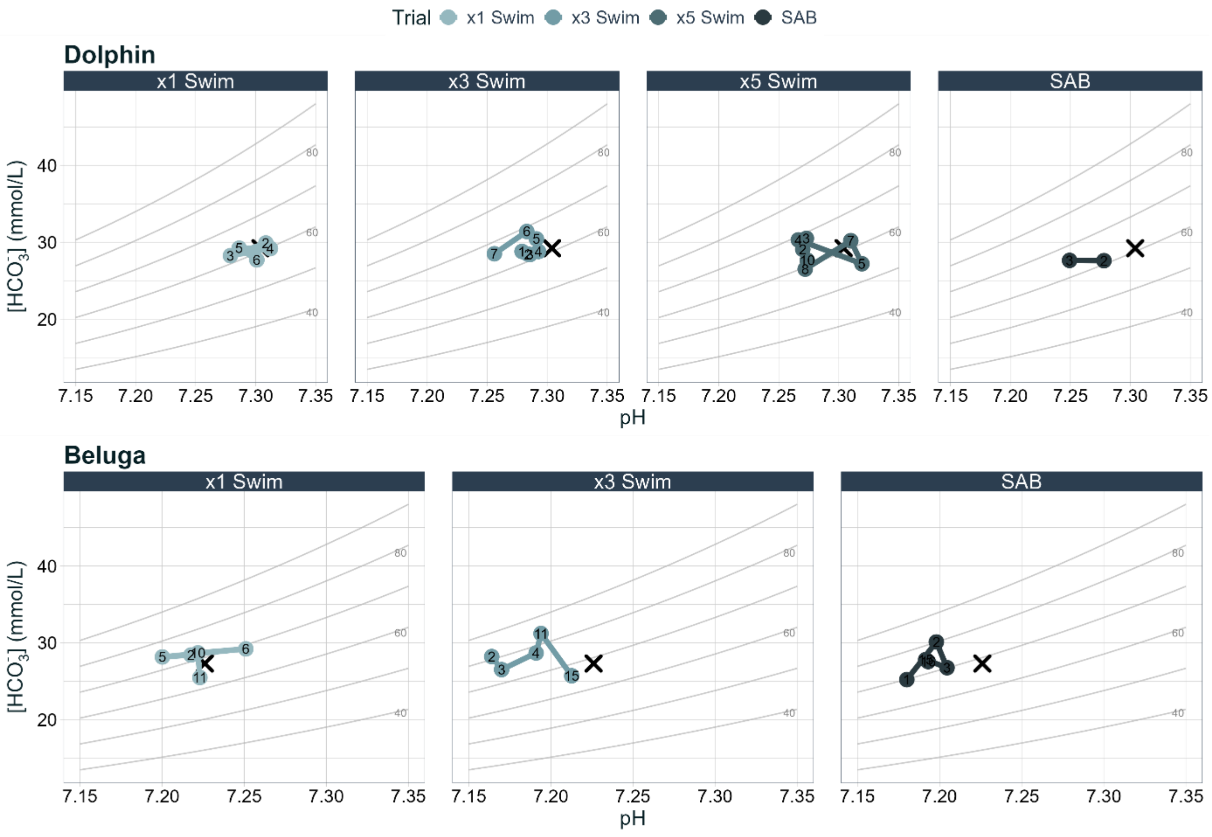

Fig. S3. Davenport diagram showing bicarbonate (HCO_3^-) as a function of pH. Points represent mean values for the nearest minute blood was sampled. Numbers on each plot represent the nearest minute the sample was taken and the lines connect the points in the order they were sampled. Data is separated by species and exercise type. The "x" on each panel represents the mean species-specific resting value. Grey lines represent carbon dioxide isobars (pCO2; mmHg).

Table S1. Sample sizes across species and animal ID per trial (resting, repetitive swims, and surface-active behaviors (SAB)) and metric type. Here, n represents each session data was collected with a given animal.

**Species**	**Metric**	**Animal ID**	**Trial type**	**n**
Beluga	Respiratory gases	Maple	Rest	2
Beluga	Respiratory gases	Maple	x3 Swim	2
Beluga	Respiratory gases	Maple	SAB	2
Beluga	Respiratory gases	Qinu	Rest	2
Beluga	Respiratory gases	Qinu	x3 Swim	3
Beluga	Respiratory gases	Qinu	SAB	3
Dolphin	Respiratory gases	Donley	Rest	10
Dolphin	Respiratory gases	Donley	x1 Swim	6
Dolphin	Respiratory gases	Donley	x3 Swim	5
Dolphin	Respiratory gases	Donley	x5 Swim	5
Dolphin	Respiratory gases	Donley	SAB	2
Dolphin	Respiratory gases	Rain	Rest	5
Dolphin	Respiratory gases	Rain	x1 Swim	6
Dolphin	Respiratory gases	Rain	x3 Swim	3
Dolphin	Respiratory gases	Rain	x5 Swim	5
Dolphin	Respiratory gases	Rain	SAB	2
Beluga	Blood gases	Maple	Rest	2
Beluga	Blood gases	Maple	x1 Swim	6
Beluga	Blood gases	Maple	x3 Swim	4
Beluga	Blood gases	Maple	SAB	4
Beluga	Blood gases	Qinu	Rest	2
Beluga	Blood gases	Qinu	x1 Swim	6
Beluga	Blood gases	Qinu	x3 Swim	5
Beluga	Blood gases	Qinu	SAB	4
Beluga	Blood gases	Nunavik	x1 Swim	4
Beluga	Blood gases	Whisper	Rest	2
Beluga	Blood gases	Whisper	x1 Swim	5
Dolphin	Blood gases	Donley	Rest	2
Dolphin	Blood gases	Donley	x1 Swim	9
Dolphin	Blood gases	Donley	x3 Swim	8
Dolphin	Blood gases	Donley	x5 Swim	10
Dolphin	Blood gases	Donley	SAB	2
Dolphin	Blood gases	Rain	Rest	2
Dolphin	Blood gases	Rain	x1 Swim	7
Dolphin	Blood gases	Rain	x3 Swim	8
Dolphin	Blood gases	Rain	x5 Swim	10
Dolphin	Blood gases	Rain	SAB	2
Beluga	Ventilation	Maple	Rest	1
Beluga	Ventilation	Maple	x3 Swim	2
Beluga	Ventilation	Maple	SAB	2
Beluga	Ventilation	Qinu	Rest	2
Beluga	Ventilation	Qinu	x3 Swim	3
Beluga	Ventilation	Qinu	SAB	3

Journal of Experimental Biology • Supplementary information

Dolphin	Ventilation	Donley	Rest	10
Dolphin	Ventilation	Donley	x1 Swim	6
Dolphin	Ventilation	Donley	x3 Swim	3
Dolphin	Ventilation	Donley	x5 Swim	5
Dolphin		Donley	SAB	2
Dolphin	Ventilation	Rain	Rest	9
Dolphin	Ventilation	Rain	x1 Swim	6
Dolphin	Ventilation	Rain	x3 Swim	5
Dolphin	Ventilation	Rain	x5 Swim	5
Dolphin	Ventilation	Rain	SAB	2
Beluga	Vasculature	Maple	Rest	7
Beluga	Vasculature	Maple	x1 Swim	2
Beluga	Vasculature	Maple	x3 Swim	2
Beluga	Vasculature	Maple	SAB	2
Beluga	Vasculature	Qinu	Rest	6
Beluga	Vasculature	Qinu	x1 Swim	2
Beluga	Vasculature	Qinu	x3 Swim	2
Beluga	Vasculature	Qinu	SAB	2
Beluga	Vasculature	Nunavik	Rest	6
Beluga	Vasculature	Nunavik	x1 Swim	2
Beluga	Vasculature	Nunavik	x3 Swim	2
Beluga	Vasculature	Nunavik	SAB	2
Beluga	Vasculature	Whisper	Rest	3
Beluga	Vasculature	Whisper	x1 Swim	2
Dolphin	Vasculature	Donley	Rest	5
Dolphin	Vasculature	Donley	x1 Swim	6
Dolphin	Vasculature	Donley	x3 Swim	5
Dolphin	Vasculature	Donley	x5 Swim	7
Dolphin	Vasculature	Donley	SAB	2
Dolphin	Vasculature	Rain	Rest	5
Dolphin	Vasculature	Rain	x1 Swim	5
Dolphin	Vasculature	Rain	x3 Swim	5
Dolphin	Vasculature	Rain	x5 Swim	5
Dolphin	Vasculature	Rain	SAB	2

Journal of Experimental Biology • Supplementary information

Table S2. Respiratory gas metabolic rates and surface recovery times by trial type (rest, swim repetitions, and surface-active behaviors (SABs). We have also calculated the mean respiratory exchange ratio (RER) for each species and trial type. The sample size (n) represents the number of data trials.

		Resting rates (ml/kg*min)					Exercise cost rates (ml/kg*min)					Return to Rest (min)				
**Species**	**Trial**	**Mean rest VO_2**	**s.d. rest VO_2**	**Mean rest VCO_2**	**s.d. rest VCO_2**	**RER rest**	**Mean swim VO_2**	**s.d. swim VO_2**	**Mean swim VCO_2**	**s.d. swim VCO_2**	**RER swim**	**Mean O_2 recovery**	**s.d. O_2 recovery time**	**Mean CO_2 recovery**	**s.d. CO_2 recovery time**	**n**
Beluga	Rest	3.12	0.87	2.54	0.65	0.82	3.12	0.87	2.54	0.65	NA	NA	NA	NA	NA	4
Beluga	x3 Swim	3.25	0.95	2.56	0.48	0.81	12.76	4.91	9.14	4.50	0.69	7.64	1.36	7.71	1.41	5
Beluga	SAB	4.10	1.13	4.04	1.22	0.98	6.68	1.16	5.55	1.14	0.83	8.41	0.85	8.54	0.95	5
Dolphin	Rest	5.40	0.71	3.91	0.72	0.723	5.40	0.71	3.91	0.72	NA	NA	NA	NA	NA	15
Dolphin	x1 Swim	5.58	0.84	4.45	0.87	0.80	7.04	1.46	4.50	1.57	0.63	2.53	0.48	2.60	0.41	12
Dolphin	x3 Swim	5.16	0.41	4.12	0.61	0.80	7.05	1.39	4.66	1.16	0.66	3.22	0.48	3.03	0.53	8
Dolphin	x5 Swim	5.38	0.48	4.28	0.81	0.79	6.27	1.55	3.95	1.27	0.64	3.41	0.76	3.41	0.71	10
Dolphin	SAB	5.38	0.17	5.26	0.89	0.98	10.56	0.58	8.30	0.45	0.79	6.44	2.22	6.58	2.37	4

Journal of Experimental Biology • Supplementary information

Table S3. Blood gas summary statistics by trial type (rest, swim repetitions, and surface-active behaviors (SABs). Lactate, HCO_3, and TCO_2 are all in mmol/L. pO_2, pCO_2 are in mmHg. sO_2 is a percent. The sample size (n) represents the number of blood samples.

**Species**	**Trial**	**Mean lactate**	**s.d. lactate**	**Mean pO_2**	**s.d. pO_2**	**Mean pCO_2**	**s.d. pCO_2**	**Mean pH**	**s.d. pH**	**Mean HCO_3**	**s.d. HCO_3**	**Mean TCO_2**	**s.d. TCO_2**	**Mean sO_2**	**s.d. sO_2**	**n**
Beluga	Rest	1.51	0.68	44.17	5.64	65.65	3.61	7.23	0.024	27.30	1.65	29.17	1.72	68.33	8.48	6
Beluga	x1 Swim	1.76	0.87	35.05	8.44	68.91	7.56	7.22	0.028	27.88	2.26	30.10	2.43	51.86	15.55	21
Beluga	x3 Swim	2.76	0.59	34.89	9.60	71.88	7.98	7.19	0.022	27.67	3.03	29.89	3.30	49.56	17.67	9
Beluga	SAB	2.61	0.62	38.63	7.39	70.99	4.66	7.19	0.014	27.44	2.38	29.38	2.39	57.38	12.14	8
Dolphin	Rest	0.55	0.22	54.00	25.65	58.95	4.32	7.30	0.021	29.23	1.10	31.00	1.41	75.75	14.73	4
Dolphin	x1 Swim	0.92	0.41	43.19	11.33	58.93	3.09	7.30	0.028	28.98	1.68	30.69	1.62	68.44	17.68	16
Dolphin	x3 Swim	1.14	0.57	43.73	15.69	61.51	4.57	7.29	0.023	29.29	2.48	31.19	2.59	66.81	14.28	16
Dolphin	x5 Swim	1.29	0.73	38.60	12.09	60.96	6.74	7.28	0.030	28.7	2.09	30.55	2.28	60.25	16.69	20
Dolphin	SAB	1.37	0.89	31.75	8.66	62.25	6.55	7.26	0.027	27.63	1.34	29.50	1.73	49.50	18.88	4

Table S4. Ventilation summary statistics for each metric: breath frequency, breath duration, and the inter-breath interval (IBI). Summary statistics were calculated in the first 2.5 min following the behavior to capture the peak change that occurred following the swim repetitions or surface-active behaviors (SAB). The sample size (n) represents the number of data trials.

Species	Trial type	Mean breath frequency (breaths/min)	s.d. breath frequency (breaths/min)	Mean breath duration (s)	s.d. breath duration (s)	Mean IBI (s)	s.d. IBI (s)	n
Beluga	Rest	8.01	1.31	1.41	0.56	6.36	4.00	3
Beluga	x3 Swim	6.90	2.89	1.61	0.45	6.56	5.57	5
Beluga	SAB	6.92	1.39	1.06	3.11	7.76	5.59	5
Dolphin	Rest	2.31	0.69	0.65	0.25	24.67	13.30	19
Dolphin	x1 Swim	4.46	1.75	0.77	0.26	19.14	13.90	12
Dolphin	x3 Swim	2.57	0.74	0.76	0.28	16.52	11.83	8
Dolphin	x5 Swim	2.27	0.68	0.72	0.30	15.53	11.84	10
Dolphin	SAB	3.85	2.21	0.79	0.31	14.63	8.10	4

Journal of Experimental Biology • Supplementary information

Table S5. Vasculature summary statistics for each metric: mean surface temperature, maximum surface temperature, and percent area perfused. The sample size (n) represents the number of data trials (rest, swim repetitions, and surface-active behaviors (SABs).

**Species**	**Trial**	**Mean surface temperature (°C)**	**s.d. surface temperature (°C)**	**Mean maximum surface temperature (°C)**	**s.d. maximum surface temperature (°C)**	**Mean proportion vasodilation**	**s.d. proportion vasodilation**	**n**
Beluga	Rest	17.23	1.43	18.71	2.40	5.69	11.86	20
Beluga	x1 Swim	17.04	1.64	18.14	2.88	3.09	8.72	8
Beluga	x3 Swim	17.74	1.62	19.83	2.90	10.19	17.96	6
Beluga	SAB	18.01	0.90	20.31	2.51	14.42	19.01	6
Dolphin	Rest	23.60	1.04	31.30	4.47	17.94	9.76	10
Dolphin	x1 Swim	24.02	1.70	31.28	4.52	24.09	19.12	11
Dolphin	x3 Swim	22.86	0.78	29.50	3.41	17.93	7.87	10
Dolphin	x5 Swim	22.67	0.96	24.52	2.44	6.57	8.36	12
Dolphin	SAB	22.07	0.82	23.53	1.75	2.40	3.50	4

Journal of Experimental Biology • Supplementary information
